# Evaluation of Effect of Curcumin on Psychological State of Patients with Pulmonary Hypertension by Magnetic Resonance Image under Deep Learning

**DOI:** 10.1155/2021/9935754

**Published:** 2021-07-26

**Authors:** Tingting Ma, Ziyuan Ma, Xiuping Zhang, Fubo Zhou

**Affiliations:** ^1^Department of Public Health, Mudanjiang Medical University, Mudanjiang 157011, China; ^2^Department of Pharmacology, Mudanjiang Medical University, Mudanjiang 157011, China

## Abstract

This research aimed to evaluate the right ventricular segmentation ability of magnetic resonance imaging (MRI) images based on deep learning and evaluate the influence of curcumin (Cur) on the psychological state of patients with pulmonary hypertension (PH). The heart MRI images were detected based on the You Only Look Once (YOLO) algorithm, and then the MRI image right ventricle segmentation algorithm was established based on the convolutional neural network (CNN) algorithm. The segmentation effect of the right ventricle in cardiac MRI images was evaluated regarding intersection-over-union (IOU), Dice coefficient, accuracy, and Jaccard coefficient. 30 cases of PH patients were taken as the research object. According to different treatments, they were rolled into control group (conventional treatment) and Cur group (conventional treatment + Cur), with 15 cases in each group. Changes in the scores of the self-rating anxiety scale (SAS) and self-rating depression scale (SDS) of the two groups of patients before and after treatment were analyzed. It was found that the average IOU of the heart target detection frame of the MRI image and the true bounding box before correction was 0.7023, and the IOU after correction was 0.9016. The Loss of the MRI image processed by the CNN algorithm was 0.05, which was greatly smaller than those processed by other algorithms. The Dice coefficient, Jaccard coefficient, and accuracy of the MRI image processed by CNN were 0.89, 0.881, and 0.994, respectively. The MRI images of PH patients showed that the anterior wall of the right ventricle was notably thickened, and the main pulmonary artery was greatly widened. After treatment, the SAR and SDS scores of the two groups were lower than those before treatment (*P* < 0.05), and the SAR and SDS scores of the curcumin group were lower than those of the control group (*P* < 0.05). To sum up, the right ventricular segmentation ability of MRI images based on deep learning was improved, and Cur can remarkably alleviate the psychological state of PH patients, which provided a reference for the diagnosis and treatment for PH patients.

## 1. Introduction

Pulmonary hypertension (PH) is a pathophysiological syndrome characterized by increased pulmonary circulatory resistance and pressure, which causes right heart insufficiency and causes premature death of patients [1]. PH treatment drugs mainly include prostacyclin analogs, endothelin receptors, and antagonists, which have a positive impact on the prognosis of PH [2]. Curcumin (Cur) reduces aortic pressure and improves left ventricular remodeling by inhibiting Na^+^-K^+^ ATPase in improving cardiac function [3]. Commonly used methods for PH detection include chest X-ray, computed tomography (CT), cardiac magnetic resonance (CMR), right heart catheterization, and lung ventilation and perfusion. The chest X-ray film is easily affected by breathing during the detection process, and the measurement error is relatively large [4]. Multislice spiral CT is less accurate than MRI in measuring right ventricular ejection fraction [5]. Right heart catheterization is the gold standard for the diagnosis of PH, but it is an invasive examination and has certain risks [6]. Cardiac magnetic resonance imaging (CMRI) can directly assess the volume, shape, and function of the right ventricle and can provide pulmonary hemodynamic parameters in a noninvasive way. It has become an ideal method for noninvasive evaluation of the structure and function of the right ventricle and has significant advantages in the diagnosis of PH.

The function of the right ventricle (RV) can be realized by segmentation of the right ventricle, but its myocardial wall is thin, adjacent to fat, papillary muscle and other tissues, and individual structures vary greatly. In addition, partial volume effects and cardiac motion often cause poor signal-to-noise ratios in CMRI short-axis movie images. The YOLO algorithm is widely used in target detection because of its fast detection speed and simple operation. Common right ventricular image segmentations include threshold segmentation, region growing segmentation algorithm, and level set segmentation. Among which, the level set segmentation process is complicated, and the amount of calculation is large, which leads to a longer operation time [7]. The segmentation effect of the region growing segmentation algorithm has been improved a lot compared with the threshold segmentation algorithm, but it is affected by the relaxation of the heartbeat during the heart image acquisition process, which leads to its poor robustness. Due to the poor contrast between the blood pool of the right ventricle and the myocardium and the large structural variation, the traditional segmentation is not very effective in the segmentation of the right ventricle. With the progression of computer technology in recent years, segmentation technology based on CNN algorithm has gradually been applied to medical image processing. Zabihollahy et al. [8] adopted CNN algorithm to realize automatic heart target detection and right ventricular initialization segmentation, then utilized a deformable model to optimize segmentation, and finally improved the accuracy and robustness of segmentation. Some researchers adopted the FCN-8 network to segment the ventricles and found that the network had a good segmentation effect on the left ventricle of the heart, with a Dice score of 0.93, while the Dice score of the right ventricle segmentation was only 0.87 [9].

In summary, although the CNN algorithm was applied to the segmentation of the right ventricle, it needed to be further optimized in clinical adoption. Based on the function of Cur, it was speculated that it may reduce PH and improved right heart function, and its effect on the psychological state of patients with PH has not been reported. In this research, the PH patients diagnosed with right heart catheterization were taken as the research object. The segmentation algorithm was optimized based on the CNN algorithm, which was then applied to the segmentation of MRI images of PH patients to evaluate the influence of Cur on the psychological state of PH patients, so as to provide a reference for the diagnosis and treatment of PH.

## 2. Materials and Methods

### 2.1. Research Objects and Grouping

Thirty patients with PH who were diagnosed with right heart catheterization in hospital from June 2019 to September 2020 were taken as the research object, all of which underwent MRI examination. There were 5 males and 25 females. The age range of the patients was 18–75 years old, and the average age was 38.62 ± 7.35 years old. According to different treatments, they were rolled into a control group (conventional antidepressant treatment) and a Cur group (Cur + conventional treatment), with 15 cases in each group. Inclusion criteria: I, patients diagnosed in accordance with the PH diagnosis and treatment guidelines of the European Society of Cardiology; II, patients with chronic thromboembolic PH; III, patients not receiving treatment with prostacyclin and endothelin receptor antagonists. Exclusion criteria: I, patients with coronary heart disease, valvular heart disease, chronic obstructive pulmonary disease, or other cardiopulmonary diseases; II, patients with severe obstructive lung disease; III, patients with severe liver and kidney damage. The process had been approved by the ethics committee of the Hospital, and all subjects included in the study had signed informed consent forms.

### 2.2. Establishment of a Heart Target Detection Model Based on YOLO Algorithm

MRI images of different organs are similar. The MRI images acquired in the short-axis direction of the heart included the heart of the region of interest and other organ structures of the body. Similar interference signals of different organs would cause a certain degree of difficulty in segmentation [10]. In this research, the heart target detection method was established based on the YOLO algorithm. For each MRI image, the horizontal and vertical coordinates *x*_min_ and *y*_min_ of the upper left point of the target area and the horizontal and vertical coordinates *x*_max_ and *y*_max_ of the lower right point were calculated according to the gold standard. Then, there were the following equations.

(1) $$\begin{array}{c} x=\frac{\left(x_{\min}+x_{\max}\right)}{2},\\ y=\frac{\left(y_{\min}+y_{\max}\right)}{2},\\ w=x_{\max}-x_{\min},\\ h=y_{\max}-y_{\min}, \end{array}$$

*x* and *y* represented the horizontal and vertical coordinates of the center point of the target area, respectively. *w* and *h* represented the width and height of the target area, respectively. The vector [*x*, *y*, *w*, *h*] was set as the gold standard for target detection and training in each MRI image.

To ensure the accuracy of the ROI, corrections were made based on the information between the MRI image sequences. The redundant detection frame in the detection result was removed according to equation (2) for the obtained position of the heart center point.

(2) $$\begin{array}{c} \left(x_{i},y_{i}\right)=\left\{\begin{array}{c} \left(x_{i},y_{i}\right),\left|x_{i}-\bar{x}\right|\leq 10,\left|y_{i}-\bar{y}\right|\leq 10,\\ \left(\bar{x},\bar{y}\right),\left|x_{i}-\bar{x}\right|>10,\left|y_{i}-\bar{y}\right|>10. \end{array}\right. \end{array}$$

In equation (2), $\left(\bar{x},\bar{y}\right)$ was the average value of the entire MRI sequence excluding the center point of the current slice of the heart.

**Figure 1:**
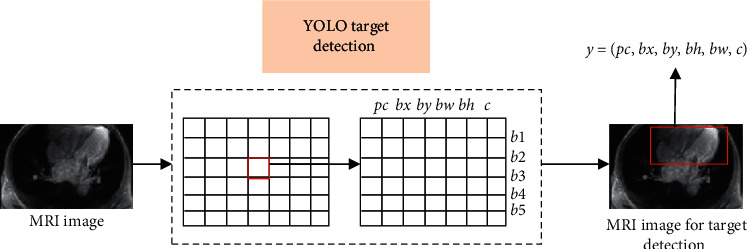
Heart detection process based on YOLO algorithm.

MRI image with a size of 416 × 416 was input and analyzed by the heart detection network of the YOLO algorithm, and a 7 × 7 grid was output. Each grid included five bounding boxes. Each bounding box contained six element vectors (*p*_*c*_, *b*_*x*_, *b*_*y*_, *b*_*w*_, *b*_*h*_, *c*), where *p*_*c*_ was the probability of detecting the heart; *b*_*w*_ and *b*_*h*_ represented the width and height of the detected heart, respectively; *b*_*x*_ and *b*_*y*_ represented the abscissa and ordinate of the center point of the heart, respectively. *c* was the confidence that the heart appeared in the bounding box. Then, the MRI image vector output by the heart detection network was (7, 7, 30). The heart detection process based on the YOLO algorithm is shown in Figure 1.

### 2.3. Establishment of Right Ventricular Segmentation Model Based on CNN Algorithm

CNN is mainly composed of convolutional layer, pooling layer, fully connected layer, and deconvolutional layer. The convolutional layer detected the local features of different positions in the input MRI image, which was expressed as the following equation:

(3) $$\begin{array}{c} C_{j}^{l}=f\left(\overset{N\left(l-1\right)}{\underset{i=1}{\sum }}C_{i}^{l-1}•k_{ij}^{l}+b_{j}^{l}\right). \end{array}$$

In equation (3), *k*_*ij*_^*l*^ was the convolution kernel, *i* was the feature map of the *l* − 1^th^ layer, *j* was the feature map of the *l*^th^ layer, *C*_*j*_^*l*^ was the feature map of the convolutional layer *l*, *C*_*i*_^*l*−1^ was the feature map of the adjacent layer *C*_*j*_^*l*^, *N*(*l* − 1) was the number of feature maps of the *l* − 1^th^ layer, • represented the convolution, *b*_*j*_^*l*^ was the bias, and *f*() was the nonlinear activation function.

The activation function is mainly for processing linear inseparable data [11]. In this research, ReLu, which had a faster calculation speed, was taken as the activation function. The ReLu activation function was expressed as equation (4), and the corresponding derivative function was expressed as equation (5).

(4) $$\begin{array}{c} f\left(x\right)=x^{+}=\max\left(0,x\right), \end{array}$$

(5) $$\begin{array}{c} f^{\prime }\left(x\right)=\left\{\begin{array}{c} x,\;x>0,\\ 0,\;x\leq 0. \end{array}\right. \end{array}$$

For MRI image *A*, *X* represented the set of all pixels in image *A*: (*x*_1_, *x*_2_,…, *x*_*n*_), and *B* represented the category (*b*_1_, *b*_2_,…, *b*_*m*−1_) included in the segmentation, and *m*=4. 1, 2, 3, and 4 represented the background, right ventricle, left ventricular myocardium, and left ventricular cavity, respectively. Then, for the pixel *x*_*i*_ of the *j*^th^ channel, the probability to correspondingly output *b*_*j*_ was as follows:

(6) $$\begin{array}{c} p\left(x_{i}=b_{j}\right)=\frac{1}{Z}\exp\left[v\left(b_{j}\right)\right]. \end{array}$$

In equation (6), *v*(*b*_*j*_) represented the value of *b*_*j*_, *Z* represented the regularization term, and the obtained predicted value *y*_*i*_ for *x*_*i*_ was expressed as *y*_*i*_=argmax[*p*(*x*_*i*_=*b*_*j*_)], and the corresponding loss function was expressed as follows:

(7) $$\begin{array}{c} \text{loss}=-\frac{1}{mn}\underset{i}{\sum }\underset{j}{\sum }y_{ij}\ln\left[p\left(x_{i}=b_{j}\right)\right]. \end{array}$$

Adam is one of the commonly used optimization methods for training neural networks [12]. Based on the CNN algorithm, Adam was employed to optimize it, and the expressions of update parameter *W* and offset *S*_dw_ and *V*_dw_ were expressed as follows:

(8) $$\begin{array}{c} V_{\text{dw}}=\alpha V_{\text{dw}}+\left(1-\alpha \right)\frac{\partial J}{\partial W},\\ S_{\text{dw}}=\alpha_{2}S_{\text{dw}}+\left(1-\alpha_{2}\right){\left(\frac{\partial J}{\partial W}\right)}^{2},\\ W=W-\beta \frac{V_{\text{dw}}}{\sqrt{S_{\text{dw}}}+\varepsilon }. \end{array}$$

*J* was the number of cycles of Adam, *α*_1_ and *α*_2_ represented the hyperparameters that controlled the average value of the two exponential weights, respectively, *β* was the learning rate, and *ε* was a very small number and was set to avoid the denominator being zero.

**Figure 2:**
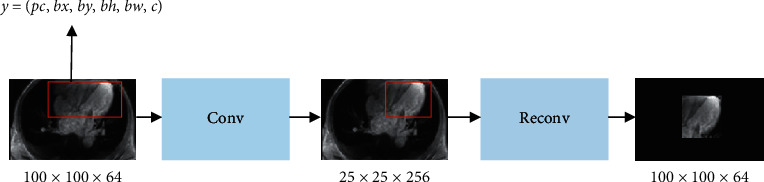
Right ventricle segmentation process based on CNN algorithm.

After the MRI image with a size of 100 × 100 × 64 processed by the heart detection of the YOLO algorithm was processed by a convolutional network (Conv) for six times, an MRI image with a size of 25 × 25 × 256 was obtained. After further six times of deconvolutional network (Reonv) processing, the MRI right ventricle segmentation image with a size of 100 × 100 × 64 was obtained. The right ventricle segmentation process based on CNN algorithm is shown in Figure 2.

### 2.4. Quality Evaluation of MRI Image Based on Deep Learning Processing

The target detection algorithm was evaluated regarding intersection-over-union (IOU). IOU refers to the area of the intersection of the predicted boundary and the actual boundary compared to the area of the union. The higher the IOU, the more accurate the target detection bounding box.

The shape of the right ventricle of the heart is mostly variable and irregular. Therefore, the segmentation effect of the right ventricle of the heart was evaluated regarding the Dice coefficient, accuracy, and the Jaccard coefficient. The Dice coefficient refers to a measure of the overlap between the segmentation result and the gold standard area. The calculation of the Dice coefficient was as follows:

(9) $$\begin{array}{c} \text{Dice}\left(A,B\right)=2\times \frac{A\cap B}{A+B}=2\frac{\text{TP}}{\text{TP}+\text{FP}+\text{TP}+\text{FN}}. \end{array}$$

In equation (9), *A* represented the standard value segmented by the doctor, and *B* represented the predicted value segmented by the CNN model. TP meant that the segmentation result and the gold standard result were both true, that is, true positive. FP meant that the segmentation result was false, but the gold standard results were all true. FN meant that the segmentation result was true, but the gold standard results were all false. The smaller the Dice coefficient, the larger the gap between the predicted result and the actual result.

The equation for calculating accuracy was as follows:

(10) $$\begin{array}{c} \text{Acc}=\frac{\text{TP}}{\text{TP}+\text{FN}}\times 100\%. \end{array}$$

Jaccard coefficient reflected the size of the difference between the data, which represented the intersection of the predicted value of the neural network and the standard value provided by the expert. The calculation was as follows:

(11) $$\begin{array}{c} JSC=\frac{\left|X\cap Y\right|}{\left|X\cup Y\right|}\times 100\%. \end{array}$$

In equation (11), *X* represented the right ventricular area predicted by CNN, and *Y* represented the right ventricular area delineated by the doctor.

### 2.5. Treatment Method and Evaluation Index of Pulmonary Hypertension

Patients in control group received conventional treatment such as oxygen inhalation, anticoagulation, cardiac strengthening, and diuresis. Patients in the Cur group were treated with Cur based on the conventional treatment, and the initial dose of Cur was 60 mg/d. According to the condition of the disease, the dose was gradually increased to 120 mg/d within 0–3 months. The treatment course of both groups was 3 months.

The self-rating anxiety scale (SAS) and self-rating depression scale (SDS) were adopted to evaluate the psychological state of patients before and after the intervention. SAS was used to evaluate the subjective feelings of anxiety patients. It consists of 20 items and is scored according to the grade of 1 to 4. “1” means no or very little time; “2” means a small part of the time; “3” means a lot of time; “4” means most of or all the time. It was scored as 4∼1 for reverse score. SDS was mainly used to assess the frequency of each symptom, using a 4-level score. “1” means no or very little time; “2” means a small part of the time; “3” means a lot of time; “4” means most of or all the time. The scoring standard: *S* = 1.25 × *T*, where *S* was the standard score and *T* was the total rough score. The lower the score, the milder the patient's symptoms.

### 2.6. Statistical Methods

The test data processing was carried out using SPSS19.0. Mean ± standard deviation ($\bar{x}\pm s$) was how measurement data were expressed, and the comparison of the mean between each group was performed by *t*-test. Percentage (%) was how count data were expressed, and the *χ*^2^ test was used. *P* < 0.05 indicated that the difference was statistically considerable.

## 3. Results

### 3.1. Analysis of Heart Detection Results Based on YOLO Algorithm

**Figure 3:**
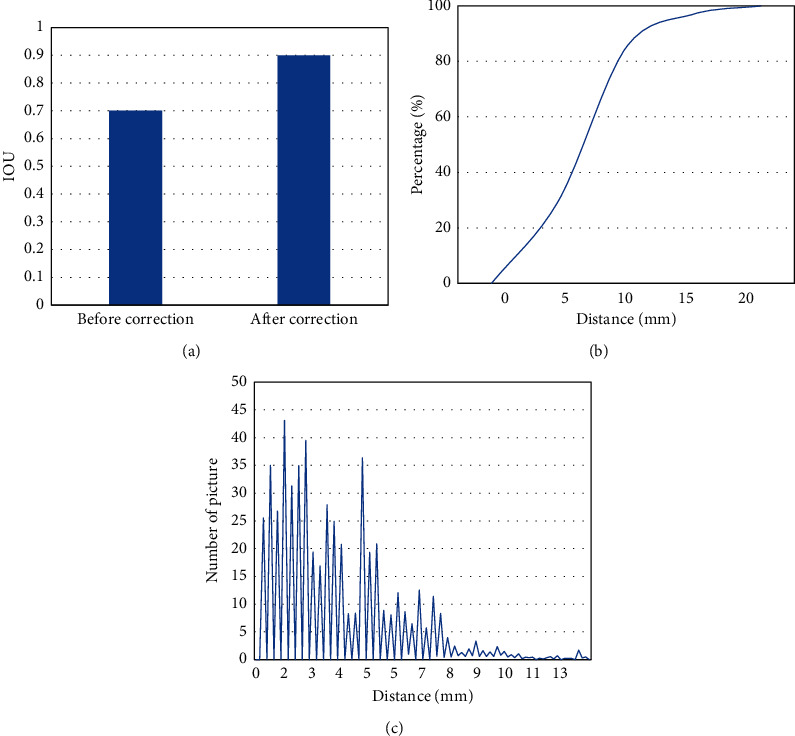
Analysis of heart detection results based on YOLO algorithm. ((a) Comparison of the IOU between the detection point and the real point before and after correction; (b) Euclidean distance distribution between the detection point and the real point; (c) percentage of the center point with the Euclidean distance less than 20 mm.).

The heart detection results before and after correction were analyzed based on the YOLO algorithm (Figure 3(a)). The average IOU of the heart target detection frame and the true bounding box before correction was 0.7023, the IOU after correction was 0.9016, and the average IOU of the heart detection frame after correction was increased by 0.19. The Euclidean distance between the center point based on the YOLO detection result and the true center point of the MRI heart was further calculated (Figure 3(b)), and the Euclidean distance between the two was distributed between 0 and 20 mm, most of which were less than 5 mm. The percentage of center points with Euclidean distance less than 20 mm was calculated, and the Euclidean distance less than 10 mm accounted for 96.4%.

### 3.2. Quality Evaluation of MRI Image Based on Deep Learning Processing

**Figure 4:**
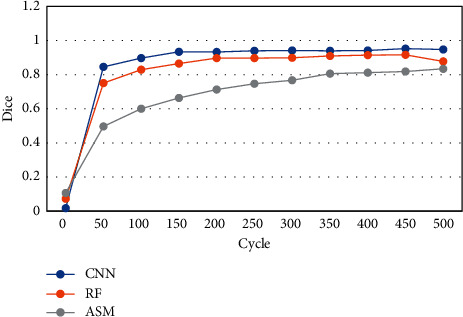
Dice coefficients based on different algorithms under different cycle periods.

Under different training cycles, the Dice coefficients of MRI images processed by CNN, active shape model (ASM), and random forests (RF) algorithms were compared (Figure 4). As the training period increased, the Dice coefficients of different algorithms showed a significant upward trend. The Dice coefficient based on the CNN algorithm was greater than that of ASM and RF under different training cycles. The Dice coefficient based on CNN algorithm was up to 0.9, then that based on ASM was up to 0.85, and that based on RF was up to 0.75.

**Figure 5:**
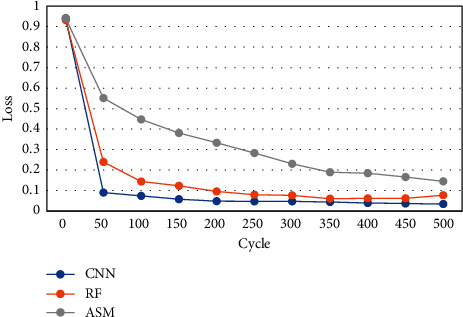
Loss values based on different algorithms under different cycle periods.

Loss value changes of MRI images processed by different algorithms under different cycle periods were compared (Figure 5). As the training period increased, the Loss values of different algorithms showed a significant downward trend. The Loss value of the proposed CNN algorithm was smaller than ASM and RF under different training cycles. When the cycle period was 100, the Loss value of the CNN algorithm was 0.05, that of ASM was 0.4, and that of RF was 0.18.

**Figure 6:**
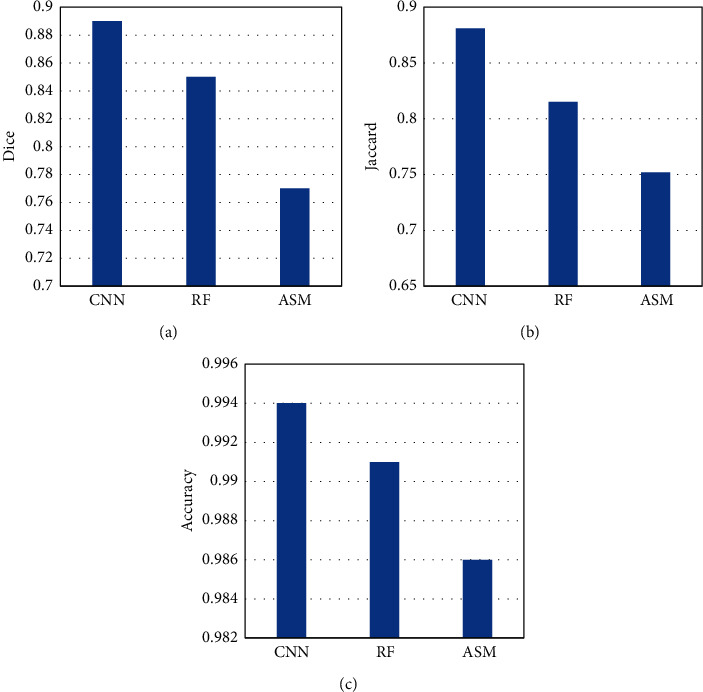
Analysis of speed-up ratio of ultrasound imaging with different algorithms.

The Dice coefficient, Jaccard coefficient, and accuracy rate of MRI images processed by different algorithms were compared (Figure 6). The Dice coefficients of CNN, RF, and ASM algorithms were 0.89, 0.85, and 0.77, respectively. The Jaccard coefficients were 0.881, 0.815, and 0.752, and the accuracy was 0.994, 0.991, and 0.986, respectively.

### 3.3. MRI Image Features of PH Patients Based on Deep Learning

**Figure 7:**
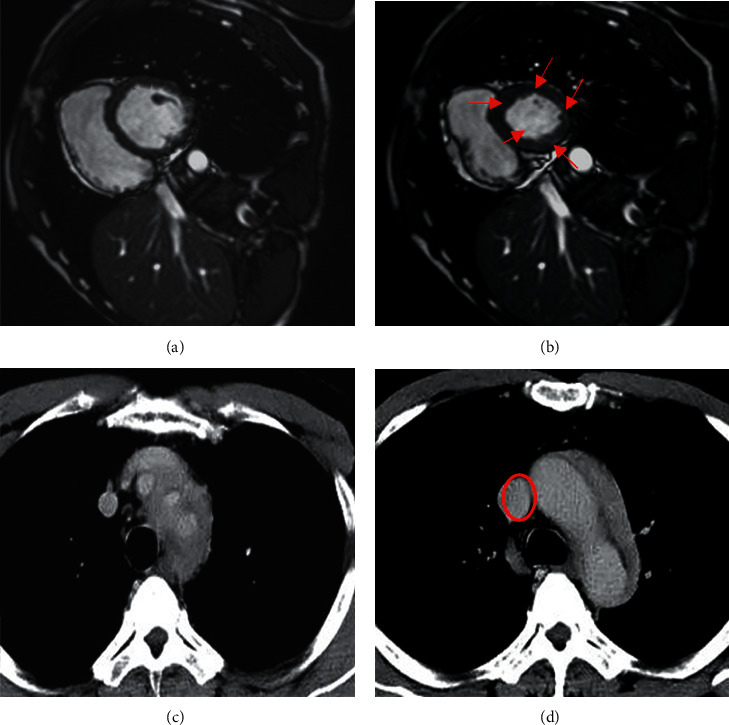
Heart MRI image manifestations. (a) Male, 28 years old, MRI image of normal right ventricle; (b) male, 30 years old, MRI image of right ventricle of PH patients; (c) female, 46 years old, MRI image of normal pulmonary aorta; (d) female, 50 years old, MRI image of pulmonary aorta of PH patients.).

The features of PH patients' MRI image based on deep learning processing were analyzed (Figure 7). In contrast to the normal anterior wall of the right ventricle (Figure 7(a)), the anterior wall of the right ventricle in PH patients was obviously thickened (shown by the red arrow) (Figure 7(b)). In contrast to the normal pulmonary aorta (Figure 7(c)), the main pulmonary artery in patients with pulmonary aortic hypertension was significantly enlarged (red area) (Figure 7(d)).

### 3.4. Comparison of Basic Data of Patients in Different Groups

A comparative analysis of the age, sex ratio, and educational background of patients in control group and the Cur group is presented in Table 1. There was no remarkable difference between the two groups of patients in age, the proportion of male patients, the proportion of junior high school educational level or above, the proportion of high school and technical secondary school educational level, and the proportion of college educational level or above (*P* > 0.05).

### 3.5. Comparison of the Psychological State of the Two Groups of Patients before and after Treatment

**Figure 8:**
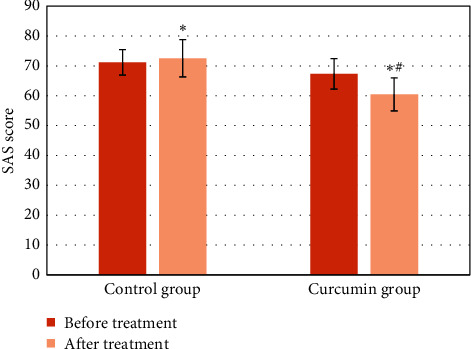
Comparison of SAS scores between the two groups before and after treatment (^∗^ means *P* < 0.05 versus that before treatment, and ^#^ means *P* < 0.05 versus that of the control group).

The SAS scores of the two groups of patients before and after treatment were compared (Figure 8). There was no considerable difference in the SAS scores of the two groups before treatment (*P* > 0.05). After treatment, the SAR scores of the two groups of patients were lower than those before treatment, and the differences were statistically significant (*P* < 0.05), and the SAR scores of patients in the curcumin group were significantly lower than those of the control group (*P* < 0.05).

**Figure 9:**
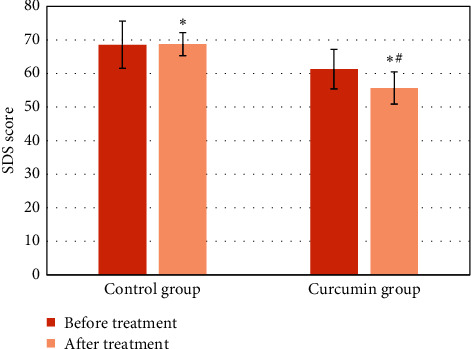
Comparison of SDS scores between the two groups before and after treatment (^*∗*^ means *P* < 0.05 versus that before treatment, and ^#^ means *P* < 0.05 versus that of control group).

The SDS score results of the two groups of patients before and after treatment were compared (Figure 9). The SDS scores of the two groups of patients before treatment were not notably different (*P* > 0.05). The SDS scores of the two groups after treatment were lower than those before treatment (*P* < 0.05), and the SDS scores of the patients in Cur group were remarkably inferior to those of control group (*P* < 0.05).

## 4. Discussion

The YOLO algorithm runs faster and is suitable for cardiac MRI images with relatively complex structures [13]. Based on the advantages of the YOLO algorithm, the information between cardiac MRI image sequences was corrected in this research. The results showed that the average IOU of the heart target detection frame and the true bounding box before correction was 0.7023, the IOU after correction was 0.9016, and the average IOU of the heart detection frame after correction increased by 0.19. Euclidean distance less than 10 mm accounted for 96.4%. These results showed that the YOLO correction algorithm can accurately determine whether the MRI image contained a heart target, and the center point of the heart area was directly obtained. The region of interest can be extracted based on the center point for further heart segmentation [14]. Dice coefficient is also called F1 score in the field of information retrieval, and it is widely utilized in verifying the effect of 3D medical image segmentation [15]. Using Dice coefficient can well characterize the segmentation effect [16]. The research results showed that the Dice coefficient of the CNN algorithm was greater than that of ASM and RF under different training cycles. The maximum Dice coefficient of the CNN algorithm was 0.9, that of ASM was 0.85, and that of RF was 0.75. The Dice coefficients of CNN, RF, and ASM algorithms were 0.89, 0.85, and 0.77, respectively, indicating that the algorithm based on deep learning (CNN) proposed had a relatively higher segmentation effect on the right ventricle, which was greatly improved relative to the results of Chen et al. [17]. The Loss value of CNN algorithm was smaller than ASM and RF under different training cycles. When the cycle period was 100, the Loss value of the CNN algorithm was 0.05, that of ASM was 0.4, and that of RF was 0.18. Such results indicated that the CNN algorithm can converge relatively faster and had a lower loss value. It was suggested that the CNN algorithm proposed can reduce the calculation works. The method of hybrid three-dimensional cavity convolution to extract long-range features was suitable for medical images, which can abstract features and had better fitting capabilities [18].

PH is a kind of psychosomatic disease. With the development of the disease, it can cause emotional and psychological fluctuations and obstacles in patients and clinically manifests as anxiety and depression. Studies suggested that targeted psychological intervention and psychological counseling for PH patients during treatment can remarkably improve the psychological state of patients and can increase the 5-year survival rate of patients [19]. In this research, no substantial difference was found in the SAR and SDS scores between the two groups of patients before treatment (*P* > 0.05). However, the SAR and SDS scores of the two groups of patients after treatment were inferior to those before treatment (*P* < 0.05), and the SAR and SDS scores of the Cur group were lower relative to control group (*P* < 0.05). It was suggested that both conventional therapy and Cur can relieve the patient's psychological state to a certain extent, while Cur can relieve the patient's psychological state to a greater degree. Cur can improve the psychological state of PH patients and can reduce their anxiety and depression. Filardi et al. [20] pointed out that the use of Cur can greatly improve the symptoms of postpartum depression in patients, but the mechanism of Cur action was very complicated, and further research was needed.

## 5. Conclusion

In this work, a heart segmentation algorithm was established based on the YOLO algorithm and the CNN algorithm, which was applied to the diagnosis of PH patients. In addition, Cur was used to treat PH patients, and the value of MRI based on deep learning in the diagnosis of PH and the influence of Cur on its psychological state were discussed. It was found that MRI based on deep learning significantly improved the right ventricular segmentation ability and increased the accuracy of PH diagnosis. Cur can obviously improve the psychological state of PH patients. However, this work still has some shortcomings that only the SAR and SDS scores are used to analyze the impact of Cur on the psychological state, while the psychological state indicators such as HAMD score and SAQ are not used for further analysis, which will be implemented in future work. In summary, the right ventricular segmentation ability of MRI images based on deep learning is improved, and Cur can greatly alleviate the psychological state of PH patients, which provides a reference for the diagnosis and treatment of PH patients.

## Figures and Tables

**Table 1:** Comparison of basic data of the two groups of patients.

Group	Control (*n* = 15)	Cur (*n* = 15)	*t*/*χ*^2^	*P*
Age (years old)	35.85 ± 3.12	39.02 ± 3.87	1.882	0.169
Male [cases, (%)]	2 (13.33)	3 (20)	2.325	0.226
Female [cases, (%)]	13 (86.67)	12 (80)	2.115	0.098
Junior high school and above [cases, (%)]	6 (40)	6 (40)	5.984	0.064
High school and technical secondary school [cases, (%)]	3 (20)	4 (26.67)	6.922	0.073
College or above [cases, (%)]	6 (40)	5 (33.33)	6.936	0.068

## Data Availability

The data used to support the findings of this study are available from the corresponding author upon request.
